# Fully-Non-Contact Masking-Based Holography Inspection on Dimensionally Responsive Artwork Materials

**DOI:** 10.3390/s8128401

**Published:** 2008-12-18

**Authors:** Vivi Tornari, Eirini Bernikola, Austin Nevin, Eleni Kouloumpi, Michalis Doulgeridis, Costas Fotakis

**Affiliations:** 1 Institute of Electronic Structure and Laser, Foundation for Research and Technology-Hellas (IESL–FORTH), P.O. Box 1527, 71110 Heraklion, Crete, Greece; E-Mails: ebern@iesl.forth.gr; austinnevin@gmail.com; fotakis@iesl.forth.gr; 2 National Gallery of Athens, Department of Conservation, Michalakopoulou 1, Athens, Greece; E-Mails: ekouloumpi@nationalgallery.gr; mdoulgeridis@nationalgallery.gr

**Keywords:** Optical Metrology, Optical Holography, Holographic Interferometry Non Destructive Testing, Speckle Interferometry, Artwork conservation, Dimensional effects, Environment, Sensors

## Abstract

Environmental control in galleries and museums is a necessity and is informed by the knowledge of ongoing processes of deterioration which can threaten the integrity and stability of artworks. Invisible dimensional changes in many works of art occur following environmental fluctuations as materials respond to the changes in humidity and temperature. The constant influence of dimensional changes usually remains invisible until displacement generates visible deterioration and irreversible damage. This paper exploits fully non contact coherent interferometry in a sequential masking procedure for visualising and studying surface deformation which is the direct effect of dimensional alterations induced by humidity changes. Surface deformation during dimensional displacements of constituent materials may occur on any artwork within an unstable environment. In this context, the presented research study explores the diagnostic potential of fully non contact sensors for the direct structural assessment of environmental effects as they occur in real time on works of art. The method is employed to characterise material responses, complementing and improving understanding of material behaviour in unstable environments.

## 1. Introduction

A long-standing debate in preservation science is the determination of appropriate safety levels of various environmental parameters within museums, all of which may influence the integrity of works of art: increases in humidity due to a large influx of museum visitors, rapid and uncontrolled fluctuations in temperature, noise and vibrations from open air concerts or organ playing, structural vibrations from passing street cars, damaging light levels during exhibition and the presence of gasses from pollution or offgassing from display cases have all been investigated and various general guidelines have been proposed for appropriate safety precautions [1-8]. In this paper, fluctuations in relative humidity have been studied, as humidity is particularly important for dimensional changes which may compromise the condition of works of art [3, 9, 10].

Many museum objects, artifacts and works of art are composed of complex hygroscopic materials (for example, painted and unpainted wood, ivory, varnishes and glues) that respond dimensionally to variations in relative humidity and temperature in order to maintain equilibrium with the surrounding environment [11-13.] Dimensional changes may occur with fluctuations in humidity; material expansion or swelling may take place with increased values of RH (and moisture content of the material) and contraction or shrinking may be provoked with decreased humidity. Swelling and shrinking of the wooden support of a panel painting, frame or sculpture can lead to the development of cracks and irreversible buckling or warping or the paint or wooden layers. Further, such dimensional changes may critically influence the strength of adhesion of the constituent layers in a painting or polychrome work of art, and may provoke subsequent loss of mechanical integrity [10-14]. As painted artworks are multilayered structures composed of heterogeneous materials with varying porosity, hygroscopicity and flexibility, with dimensional changes which follow a change in humidity, a multitude of non-uniform internally generated stresses and strains may be generated [15-18].

The amount of stress or load which a material can safely undergo before fracture is governed by Hooke's law, which forms the basis of the theory of elasticity. In brief it states that for certain ranges of stress, the strain produced is proportional to the stress applied and disappears on its removal (elastic region); whereas in the limit of proportionality the linearity ceases (plastic region), the material reaches the elastic limit (yield point), accompanied with permanent change (strain) leading to fracture even without further loading. The elastic limit has been used to define the onset of damage in works of art and related materials [17-21]. In this context, if RH fluctuations can cause shrinking or swelling stresses that exceed the elastic limit, damage is provoked and fracture is expected in case of further loading. However, even following irreversible and permanent changes (damage), effects of dimensional changes may initially be invisible [22, 23] until visible and irreversible damage occurs

The cumulative effects of dimensional displacement may present visible phenomenon which are well-known to conservators; for example, these can range from changes in craqueleur patterns on the surface of a varnished painting to paint losses, detachments among layers and cracks in a wooden support [24, 25]. Therefore, the exploration of displacement leading to changes and deformation at visibly imperceptible levels (i.e. prior to the occurrence of phenomena related to irreversible damage) is critical for technology innovation in preventive conservation.

In this paper, fundamental research on direct measurements of dimensional responses of model samples which follow induced humidity fluctuations is presented. Measurements can be used to determine dimensional changes on a micro-scale using non contact laser instrumentation based on Optical Holographic interferometry principles and results suggest that these measurements can further the understanding of mechanisms of damage and deterioration. The perceptible changes that eventually occur, and the material responses that vary, can be studied in order to distinguish between phenomena which result from natural ageing from those related to or aggravated by climate-induced deformation. Ideally, Optical Holographic interferometry can be used to inform risk assessment of individual objects, to predict specific future deterioration, and for the establishment of realistic environmental values based on concrete data for museum galleries.

In this work, Optical Holographic interferometry Non Destructive Testing (HINDT) methods are proposed for revealing small material displacements in the range of few to some tens of micrometers and for identifying hidden flaws or heterogeneities in dimensional response as well as damage or the onset of plastic deformation (or fatigue) [26-29]. The technique is completely non-invasive, nondestructive and fully non-contact, allowing the examination and assessment of any object regardless of size or fragility; therefore, the methods can routinely be used to analyse art works and sensitive historic artifacts for incipient faults, the onset of deterioration, or the assessment of long-term damage. Hence HINDT allows the adoption of corrective measures at an early stage, or can inform treatment of damaged objects [30-37]. This method represents an alternative and significant improvement to the use of other displacement sensors which require contact and the fixing of a sensor to the surface of a work of art [38, 39].

Moreover the direct non contact recording of deformation or displacement following small variations in the environment on works of art represents a promising field of sensor research and development of relevant instrumentations and methodologies [40-42]. In this work, the dimensional changes due to natural or imposed environmental fluctuations is exploited and used as a dimensional sensor to indicate damage levels and calibrate baseline values for structural stability under specific loads [29]. Such non contact and direct type of sensors utilizing techniques based on similar principles presented here, are expected to become essential in the study of climate change effects and may be used to discover and assess characteristic profiles of individual materials and complex artworks in order to determine behavior patterns [27, 28]. By establishing the sensitivity range of a work of art to environmental change suitable conditions to prevent further damage and reduce deterioration can be chosen. Further, analyses can be find-tuned depending on the investigation, in order to provide concrete evidence for the determination of acceptable environmental parameters for the display, transport and storage of works of art.

## 2. Experimental Description

### 2.1. Brief review of experimental technique and background

**Figure 1.**
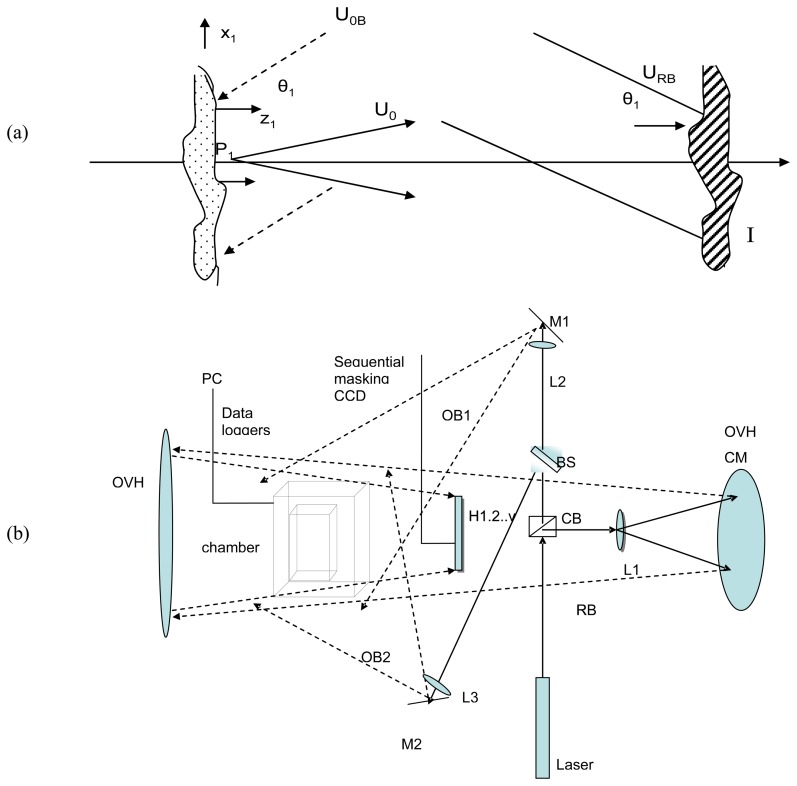

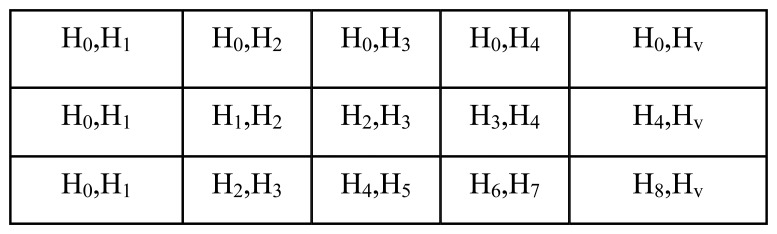
(a) A diagrammatic setup of a holographic interferometer sensitive to record out of plane (θ) displacement where U_0b_ is to the object wave, U_0_ is the surface reflection wave shown from an object point P_x.y_, U_RB_ is the reference wave beam, I is the plane of recording the intensity distribution of interference, and (b) optical geometry of experimental methodology, L (lens), M (mirror), OVH CM (Overhead collimated mirror), CBS (Cube beam splitter), BS (Beam splitter), (c) procedure of masking scheme, H: hologram, 0 is first exposure at resting position and 1-v consequent exposure combination.

In holographic recording a single laser beam is divided into two beams, one (U_0B_) directed to the subject matter and the other (U_RB_) directed to the plane of the photosensitive medium or detector as shown in Figure 1.The two beams form an intensity distribution (I_x,y_) containing the phase information of the subject as an interference pattern. The underlying principle of holographic interferometry is the sequential recording of optical wavefronts, which can later be reconstructed simultaneously.

Sequential holographic interferometry implemented here assumes the masking result of superimposing a reference H_0_ holographic record with H_1-v_ sequential records in a masking procedure to form a temporal sequence of high resolution interference patterns. In order to obtain one interferometric evaluation the subject is slightly deformed in one of the two holograms. Since the first and the second optical waves represent the light scattered by the object before and after a slight deformation, what is primarily recorded is the phase difference of these waves. The phase difference expressed in optical path displacement is related to physical quantities indicative of the deformation that the subject has undergone between the two hologram exposures [20, 27]. The description of holographic interferometry, where the two object wavefronts are recorded on the same plate, is known as double exposure holographic interferometry and is the basis for the sequential masking technique used in the presented monitoring experiments. In sequential masking holographic interferometry deformation over a duration greater than that assessed with double exposure holography is employed and therefore the relative displacement as function of time is measured [45]. The resulting permanently recorded fringe pattern contains the information about the change in shape of the detected surface of the subject during the applied time interval. The recorded displacements are of the order of only a few micrometers and therefore the technique is suitable for the determination of minute surface movement due to environmental fluctuations.

### 2.2. Experimental Methodology

The objective of the investigation is to observe dimensional changes in materials under normal conditions and typical environmental variations. Such variations experienced by an object or material may occur following (a) display in a gallery, (b) removal to another gallery within a museum, and (c) loan to an exhibition in a foreign gallery with an abrupt change of climate, or even due to uncontrolled fluctuations of the internal museum environment following a change in weather conditions. In order to simulate humidity fluctuations, measurements are focused (a) on slow rates of RH change with a gradual increase in absolute humidity, (b) by a smooth or rapid change and (c) on the measurement of dimensional changes following an abrupt increase or decrease in ambient conditions.

Double exposure (DE) holographic interferometric inspection with sequential masking was used to follow dimensional changes in wood samples of Pine (Pinus Mugus) and Oak (Quercus robur) which were cut tangentially and radially. Fully conditioned wood samples were approximately 7 × 10 × 1 cm and other samples studied include both Ivory and Horn. Aluminium was used as control sample to visulise and exclude rigid-body motions in the recording system.

The samples were interchanged between two controlled conditions: within a climate box (loading) and the external environment (unloading); two digital probes were used to measure conditions within the airtight chamber as well as the external conditions (RH and temperature). Environmental conditioning was achieved by using saturated salt solutions for the establishment of different RH values. Measurements were taken using the set-up described in Figure 1, c to determine material responses as a function of the rate and degree of change in RH. Laser and Environmental parameters are outlined in Table 1.

During the loading period for simulating conditions (a)-(c), the samples were enclosed in the chamber, establishing a steady RH/T. When the sensor's reading was steady ensuring that the loading period was been established, the samples were unloaded. The unloading period was achieved either by applying a gradual or abrupt change. Sequential DE interferograms were recorded while the samples adjusted to a new environment during unloading. In (a)-(b) conditions termed Phase 1-2 respectively, the induced gradual change corresponded to a rate of change of 3–5 % RH/h and was applied to simulate changes in museum environment for 45–65 % RH, 65–55 % RH and 55–65 % RH as Phase 1, 65–45 % RH as Phase 2. In conditions termed Phase 3, the rate of change was more rapid at 5–10 % RH/h and was achieved by evacuating the chamber to simulate seasonal fluctuations in environment from 45–75 % RH and 75–45 % RH. In Phase 4, changes from 45–85 % RH and 85–45 % RH were applied. In order to achieve such an abrupt change with a rate of change exceeding 10-15% RH/h, the samples were removed from the chamber and placed within the external environment, simulating environmental changes which may occur during transportation to another country, accidental environmental alterations or outdoor exposure.

**Figure 2.**
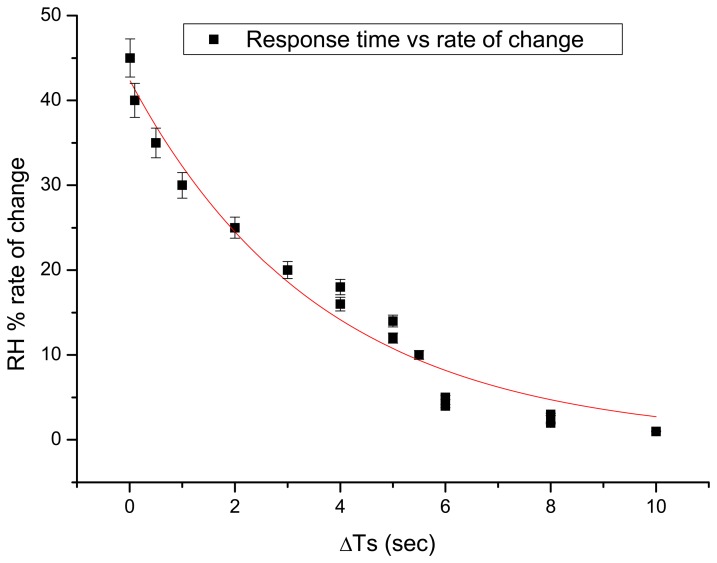
Graph indicating the dependence of the dimensional response time on the rate of RH change/ % RH.s^-1^. As the rate of change of RH increases, the response time of the sample which is required for the observation of dimensional changes decreases, indicating the need for more rapid acquisition times.

By alternative use of a continuous wave (CW) and a nanosecond pulsed laser, different durations of exposure time for a single holographic recording (one exposure) and variable intervals between exposure of the second holographic recording in a masking sequence were required in order to produce interferometric data of equilibrium responses, as shown in Figure 2. Only Phase 1, 2 was possible to be studied with the first masking arrangement (Figure 1c) while the other types of masking are essential for higher rates of change.

The experimental procedure and the evaluation of the interferometric response of the samples are indicated in Figure 2. During all phases of the measurement, the environmental (RH) conditions varied while interferometric observation was used to monitor the response of the samples. The measurement began when an induced RH was altered from an established value (loading) towards another value (unloading). From the moment the change is applied (% RH/sec), the response time (Ts) is counted following the first evidence of dimensional change (Δz/micrometers, surface displacement). It was observed that different samples require a different time-lapse prior to the first response in fringe pattern indicating the start of detectable surface displacement. Thus, the first parameter for monitoring the equilibrium process is the estimation of the characteristic response over a particular rate of RH% change. In parallel, during unloading the corresponding time interval for the recording of sequential double exposures (response time ΔTs) is different but proportional to the time required for the onset of the observation of change (Ts). The length of time from the first until the final evidence of dynamic displacement (ΔTf-ΔTs) is the total interferometric response time and is equivalent to the time required to attain equilibrium (shown later in Figure 7). Response time is the time during which a displacement generates an interference pattern in a pre-set interval between exposures. Monitoring is finished when fringe-free (no recordable displacement) DE interferograms are observed.

### 2.3 Selective use of lasers

The dimensional response to RH change is recorded in the holographic set-up (diagrammatic recording geometry in Figure 1) with alternate use of pulse or continuous wave (CW) lasers in each phase of the experiment. The time interval between the two exposures of one interferogram and the time interval between subsequent interferograms is chosen depending on the magnitude and rate of RH variation (Phases 1-3). With the CW laser gradual changes are monitored with intervals between exposures of 5–120 s and intervals between subsequent interferograms varying between 30 min and 60 min if the samples continued to change. With the pulsed laser exposure of only 15 ns, abrupt changes are monitored while materials rapidly respond, with intervals between exposures of 5–500 ns and between subsequent interferograms from 30 s to 600 s.

To assess the rate of dimensional change for interval settings the CW double exposure is applied first. If the sample undergoes rapid dimensional changes during the relatively long exposure of the CW the fringe pattern is of high frequency or irresolvable, and pulse illumination is better suited. In the same manner if masking is applied without correspondence to the rate of change again the fringe pattern is of very high or low spatial frequency, thus serving as a self-limiting control indicating the optimum experimental settings. In this case a pulsed laser with a pre-set interval between the two pulses is used to measure the time during which the dimensional change occurs, allowing the evaluation of the degree and the rate of subject-environment interaction. If the sample undergoes small dimensional changes a fringe system of low frequency and high visibility is obtainable. The CW laser can be used to reveal the entire duration required to achieve equilibrium with the surrounding environment. Since the velocity of dimensional change decreases with time, an increase in the time interval between exposures is used to provide successful monitoring; if strict isolation of the recording system can be guaranteed, the final equilibrium can be recorded even several days following the onset of unloading, depending on rate of RH change. However, by changing the time interval between exposures in CW recording the detection of slower states of reaction can be traced, thus sensing the dynamic dimensional changes.

In summary, the pulse laser enables holographic exposures of materials that change quite rapidly, the intervals between exposures being 5 s. Therefore, the double exposure pulse is used to record motion with short intervals to assess the rate of change of samples. The double exposure continuous wave is used to record motion with longer intervals to assess the time required to achieve equilibrium with the new environment. Masking scheme follows the same procedure accordingly since the H_0_ hologram can be used as the first hologram for sequential recording only if the rate of change is kept low.

## 3. Results

The cycles of loading and unloading were divided in two broad categories: (a) gradual changes where the RH change is permitted to change with a low rate and at low values (Phases 1 and 2); and (b) abrupt changes where samples are exposed suddenly to a different environment with variable conditions (Phases 3 and 4). The interferograms can be used to divide the responses of materials in two broadly different behaviour as a function of interferometric response time.

**Figure 3.**
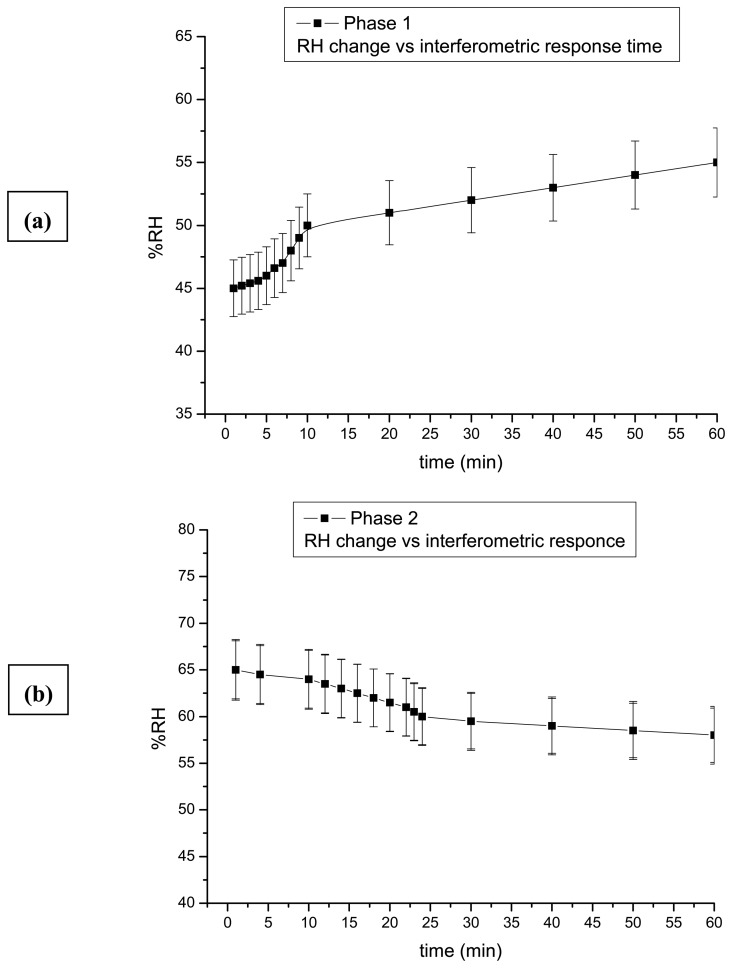
Graphs of the average total displacement response time of samples of wood and ivory during gradual RH% changes, in (a) Phase 1 displacement is smooth and requires CW exposure and longer interval time to capture interferograms and in (b) Phase 2, samples react smoothly with a similar range of CW exposure and interval times to that seen in Phase 1.

**Figure 4.**
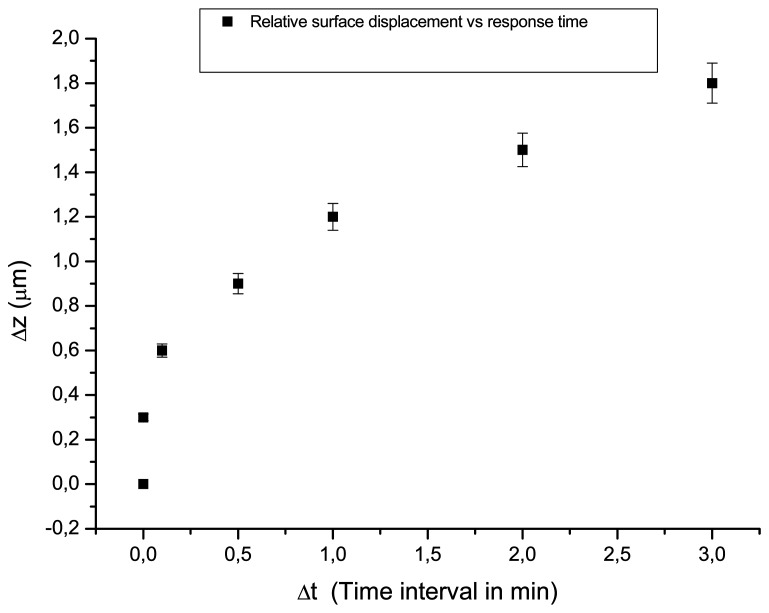
Smooth dimensional change is evident from surface displacement at specified times and relative displacement is low even following longer time intervals.

During Phases 1 and 2 (Figures 3 a,b) the values of RH are gradually changed to permit materials to adjust to new RH conditions smoothly. Indeed, the results are acquired only using the CW laser, and samples exhibit minimum displacement (only a few pairs of fringes) with maximum dimensional change reaching approximately Δz ≈ 0.6 μmmin^-1^ (Figure 4). The results reflect relative stability in this range of fluctuations. The exposure intervals involved are of critical importance since any dimensional change is limited according to the interval between exposures indicating the time within which the changes in shape of the samples occurs. Even longer intervals between holograms of 3 min revealed no substantial displacement. This indicates that the small changes of RH used provoke a slow and continuous change towards equilibrium, illustrating the relative stability of the materials within this range of rate of fluctuations.

**Figure 5.**
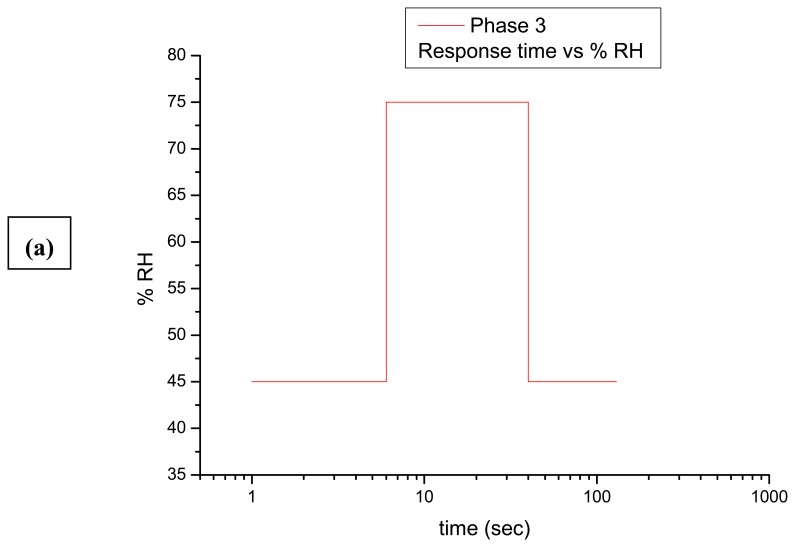

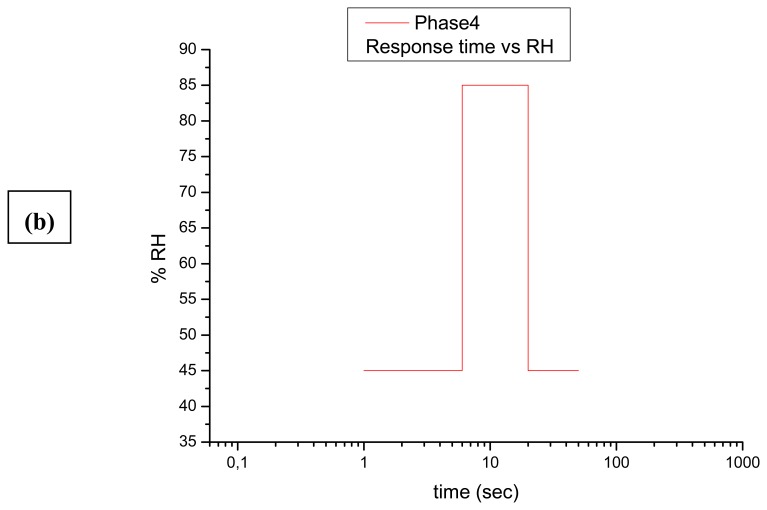
Graphs of the average displacement response during abrupt RH% changes, in samples of wood and ivory (a) Phase 3 detection of displacement requires pulsed exposure and short interval times and in (b) Phase 4 samples exhibit rapid displacement in Q-switched pulsed exposure and interval times.

During Phases 3 and 4 (Figures 5 a,b) the intervals between exposures starts from 5 s and increases to 5 min, followed with alternate use of pulsed and continuous lasers, and were continuously monitored until a fringe free interferogram was recorded towards the end of the experiment, indicating the establishment of equilibrium. The recorded DE pulse interferograms demonstrate a significantly different behaviour compared with Phases 1 and 2. An appearance of a fringe pattern of minimum displacement of approximately Δz ≈ 0.3–0.6 μm is present at an exposure interval as short as 5 s. An explanation of this behaviour is that the large difference between the RH of the unloading environment and that of the loaded sample leads to an immediate dimensional response and a rapid change in the sample volume. Results suggest that, following abrupt changes, samples absorb or loose significant moisture and thus deform during moisture loss (or gain); kinetics of change are relatively independent on the rate of change of external RH (which is immediate) and instead reflect the magnitude of the difference between the RH of loading and unloading. In Phases 3 and 4, an initial rapid dimensional change is followed by a different and slower rate of displacement (swelling or shrinking). After the first abrupt change in Phase 3, initially large values of dimensional change are followed by more gradual changes and small displacements that can last for several hours. This is an indication that the samples can accommodate or adjust to environmental changes homogeneously; following an initially rapid dimensional change upon the abrupt change in conditions, samples gradually adjust to equilibrium with small rates of dimensional changes. However, the required monitoring time between the onset of unloading to the fringe free interferogram, with the pre-set interval of 5 s, can be significant. Results suggest that, following repeated changes, radially cut wood samples simultaneously undergo loss of moisture from the edges and gain moisture towards the centre, thus modifications in the sample shape (and continual sample displacement) may occur even when the external environment ceases to change.

**Figure 6.**
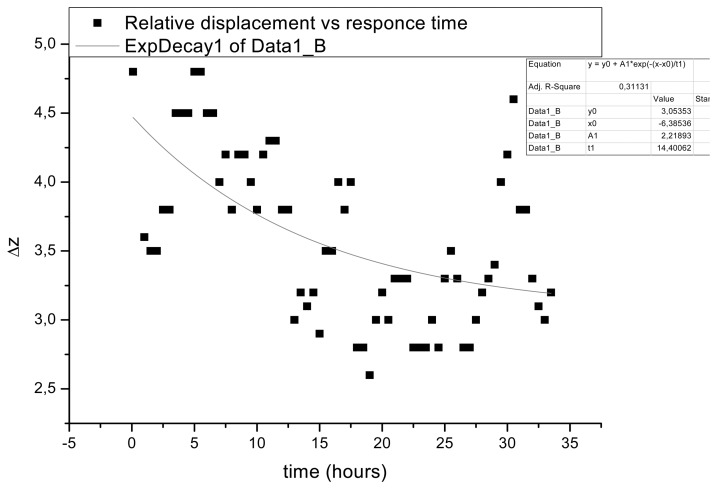
Relative displacement of samples in Phases 3 and 4 following an abrupt change in RH as a function of time. Relative displacement remains high even in shorter intervals and for extended periods of time. Equilibrium is hard to be achieved even if statistical error and extreme data points be neglected.

During Phase 4 responses of samples begin to deviate even more than those observed in the other phases. The displacement occurs over longer periods and, from the onset, is even more rapid. Interferograms are formed with small intervals and displacement can last for days (Figure 6.) Dimensional changes are apparent throughout the course of measurements revealing discontinuous motion as samples adjust to equilibrium.

**Figure 7.**
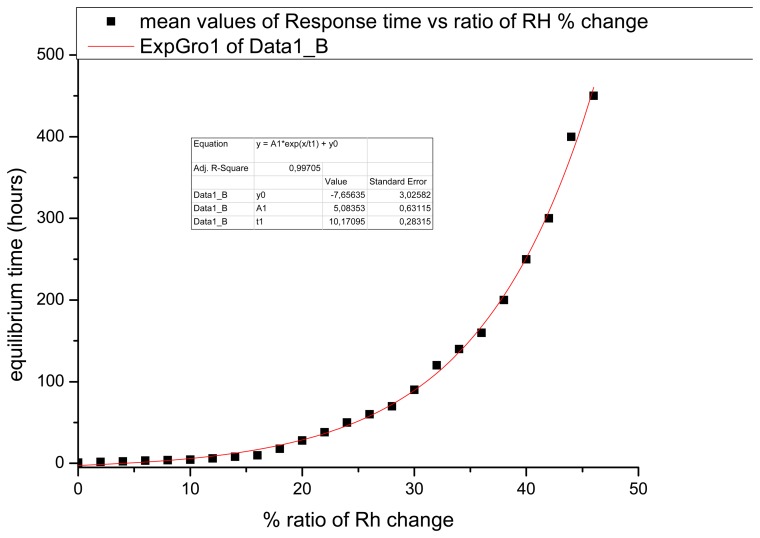
The mean equilibrium time vs. RH ratio of change demonstrating that the greater the ratio between loading and unloading conditions, the longer the time required to reach equilibrium.

Using the above parameters, the responses of different samples to environmental changes were measured. The average profile depends on the ratio of the different RH between loading and unloading indicate and experimentally prove that with greater changes in RH, more time is required to achieve equilibrium (Figure 7). The relation between the rate of change and equilibrium time (to the fringe free interferogram) is seen in Figure 7 (in the pre-set interval 5 s). The diagrams represent the response of materials which can be recorded within a specific time with the pulsed laser. The relation between rate of dimensional change and equilibrium time can be determined in a short-term investigation by holographic interferometry. although the loading time at an established RH value can vary, and the unloading can be adjusted to induce greater RH rates of change A pulsed laser is used in order to assign the pre-set time required as materials adjust towards equilibrium. As has been seen from the results the materials give an interferometric response within 5 s only when they undergo a rapid displacement. Thus the time period over which the rapid displacement occurs varies according to the applied load. The materials change shape as they swell or shrink and the measurement of displacement is correlated to their equilibrium time. Thus, the time required to attain equilibrium can be related to the dimensional change that occurs between specific periods. To measure the equilibrium time, days may be required and this can achieved using CW holographic interferometry. Thus, the duration of recording the process towards equilibrium depends on the temporal specifications of the acquisition tool.

**Figure 8.**
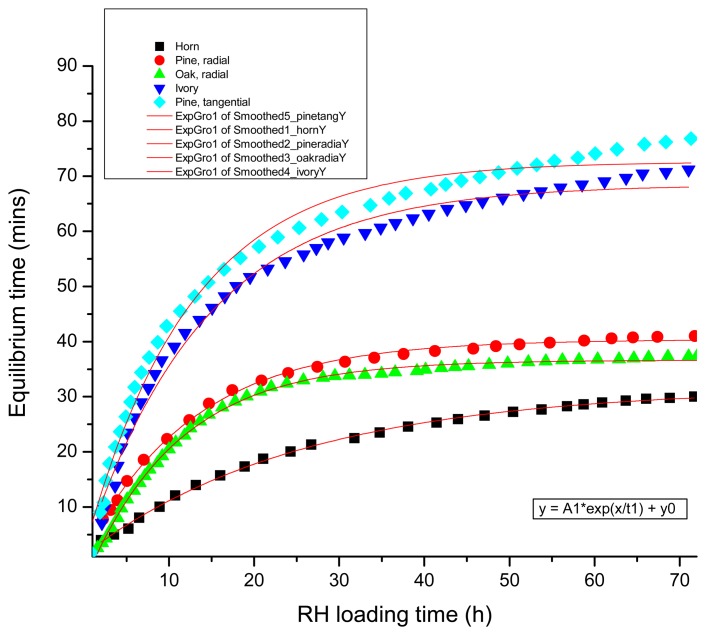
Mean equilibrium time of different materials.

Figure 8 shows the assigned equilibrium time of materials after specific periods in an established environment of RH, using the DE pulse masking technique. The long-term equilibrium time of materials can be estimated from the reaction after the first hour by extrapolation when a pulse laser is available. It is worth highlighting the behavior of the different samples indicated in Figure 8; when the difference between loading and unloading increases, the difference in relation to equilibrium time becomes smaller. The critical unloading period is shown to be the 24 h period following unloading or climate change, but between 24 to 32 h the differences are significantly reduced. After 48 h or 60 h of unloading conditions variation in dimensional response becomes even smaller. Differences in the Equilibrium time between radial and tangential cuts of wood are obvious, while differences between radially cut oak and pine are small. Similarly, horn and ivory, both bone materials, reveal different equilibrium times which is ascribed to the higher density of horn compared to more porous ivory.

### 3.1. Model painting sample

For the start-up reaction to the unload process, analysis of a model painting with digital holographic speckle pattern interferograms [29] without masking based on the out-of-plane principle described in the set-up in Figure 1, have been recorded during the first 3 min with CW laser each every 2–6 seconds and different aspects of surface movement have been revealed, as described below. Examples of interferograms from those recorded are shown, in order to demonstrate the potential of the technique to record the start-up of reaction of wooden panel paintings following changes in ambient conditions. Quantified surface deformation during first reactions to new ambient conditions and deformation plot are calculated by fringe data and numerical processing. Two stimulated conditions are shown:

a) Analysis of the Model sample with a change in RH from 75% (obtained with NaCl saturated solution) to ambient RH 44%
b) Analysis of the Model sample with a change in RH from 10.5% (obtained with silica gel) to ambient 44%.

**Figure 9.**
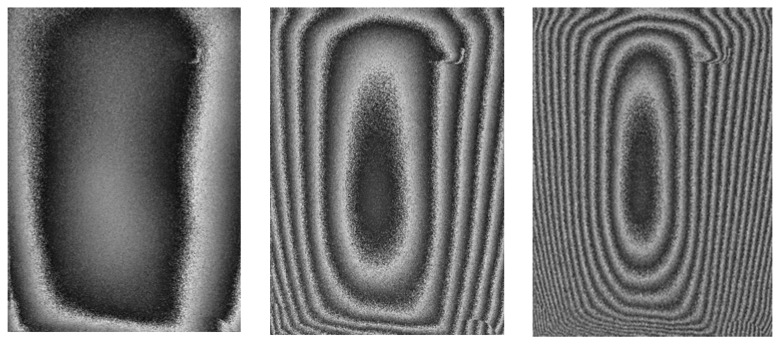
Wrapped interferograms recorded at (a, left) 8 sec, (b, centre) 120sec, (c, right) 180sec during unloading process.

For the specific sample and for both cases mentioned above ((a) and (b)) the number of fringes–and so the displacement–has been measured for a total duration of 200 sec. For both cases the velocity of changes has also been calculated (Figure 2(a)). The interferograms presented in Figure 9(a), (b), (c) represent exemplary measurements taken as the model sample adjusted from 10% to 45% at different time delays from the induced change and visualise the immediate and increasing value of reaction.

**Figure 10.**
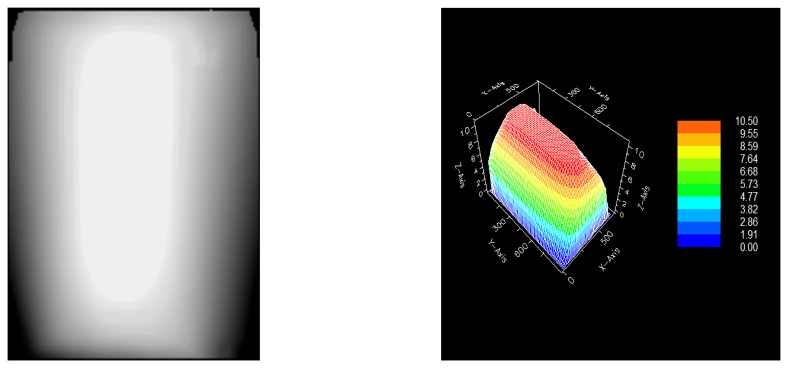
(a) Unwrapped interferogram, (b) 3D image of displacement of the entire surface.

As the fringes are symmetrical in topology terms to both directions (from right to left) it is possible to measure the fringe-pairs along one direction, eg in this case along x, to determine the relative magnitude of deformation. In non-symmetrical cases multi-viewing angles are used in both techniques. The raw interferograms are further processed following phase unwrapping procedures, to deliver the 3d plot of deformation [46, 47]. The false colour images in Figure 10 represent the unwrapped phase by interpolation and the 3D plot of deformation for the case of displacement shown in Figure 9c.

In the 3D image (Figure 10b), the y-axis corresponds to the sides of the panel, x-axis to the top and bottom and z axis to displacement, following 180 sec after climate change. The direction of displacement in z, is interactively assessed by raw data evaluation or by numerical calculation. This physical process though gives both +z, -z direction of displacement during the same experiment.

**Figure 11.**
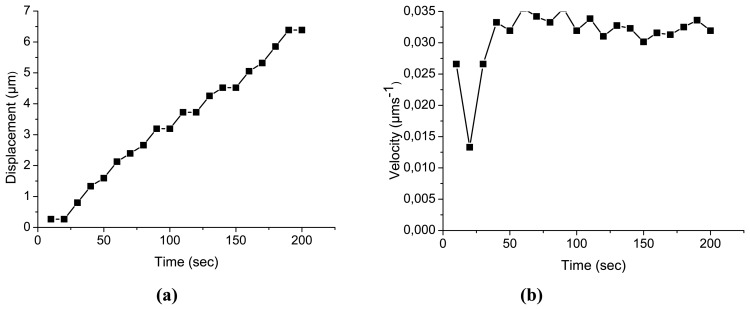

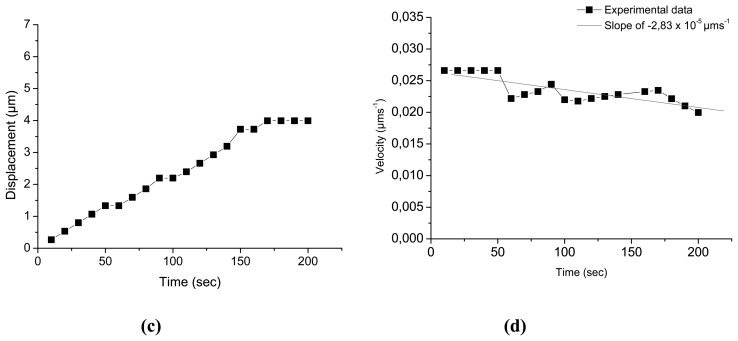
Displacement vs time graphs for RH changes, (a, top left) 75% to 45% and (c, bottom left) 10% to 45%, velocity vs time graphs for RH changes (b, top right) 75% to 45% and (d, bottom right 10% to 45%)

Due to the restrictions in image resolution encountered in the speckle decorrelation of the image it is not possible to detect fringes after the lapse of ≈200 sec. Speckle decorrelation is advanced due to the significant difference between the sample's original position and its position following the change in %RH ≈200 sec later depending on the value of change. However the technique's fast responces allow following start-up reaction in a variety of conditioning. As observed in Figure 11, where displacement vs. time is presented, in the case where the sample adjusts from dry conditions to increasing RH the total displacement is much more smooth and with lower values than those of the case in which the sample adjusts from high to a lower RH. Importantly, the immediate response even following only 50 seconds, a change of 1.5 micrometers is induced. It is also of interest that the velocity of change in the first case is more stable, but high at 0.035 μms^-1^. In contrast, in the second case a smaller velocity of 0.026 μms^-1^ decelerates at approximately 2.83 ×10^-5^ μms^-2^ to 0.2 μms^-1^ following 200 s. It should be stressed the fact that the start-up reaction is immediate and intense presupposing long-term relaxation processes towards equilibrium as has been shown already in previous sections but also presupposes for the risk of immediate damage which forms a main concern in transit exhibitions.

## 4. Discussion

During unloading two main patterns of behaviour have been revealed: (1) during the smooth changes, uniform and (2) during the abrupt experiments, non-uniform motion. The results are obtained with the DE CW and DE pulse techniques, which can record the out of plane displacement (Δz) of the object between two instants in time (Figure 4a). The change in position is distinct from the real position of any point on the object in z direction. This is because in DE the resulting fringe pattern contains all the information about the change in shape of the seen surface of the subject but does not reveal the direction of surface movement. If the nature of the displacement is unknown, as is sometimes the case for environmental change, absolute information about the direction of motion will not be a priori determined. In most cases, however, the direction of this deformation can be understood from knowledge of the material from which the subject is made and the 3d motion of the fringe systems.

Figure 9 shows an example of the information obtained using the double exposure masking technique. As can be seen, since the direction of the deformation is not directly known, the | Δz | must be used. Since the measurements were taken when the materials were not far from equilibrium and due to the smooth form of the graph, it is reasonably certain that the process (contraction) is smooth and well behaved. It can be noticed from graphs (Figure 2, for example) that the errors are of the order of a fringe pair in the interference pattern and that the Δt (time interval between exposures) is increased when Δz is small in order to record enough fringes to reduce the error. The small number of fringes present at any time indicates that the materials adjust to slow environmental changes in more or less continuous equilibrium. By sampling various points in a closed cycle Low-High-Low RH, the time required for all displacement to cease (no fringes) can be discovered as can the necessary time for the establishment of equilibrium in both extremes. The extent of distortion that occurs during the process of swelling or shrinking can also be determined. Pattern forms, if analysed, can also give an idea on how abrupt a change of RH or temperature a given subject can safely withstand, and whether and how repeated cycles have a cumulative effect on the material deformation shape.

The behavior observed in Phases 1 and 2, appears to indicate that theories of the relationship between environment and dimensional change can be tested experimentally with high sensitivity by holographic interferometry. Here, holographic interferometry has been employed to investigate the attainment of states of equilibrium and to ascertain whether equilibrium can be maintained during slow changes in environmental humidity and temperature. In Phases 3 and 4 the materials show a response of a different nature. Because the changes in the environment are too rapid for the materials to remain in equilibrium, a more irregular pattern of change occurs. The probable interpretation of this response towards equilibrium may reveal an oscillatory or discontinuous motion. The recorded displacement gives greater values of 2.8–4.9 μm within 5 s. During the attainment of equilibrium, longer intervals and shorter masking between exposures are progressively required in order to obtain a significant number of fringes. Another interesting behaviour during Phase 4 is the similar number and appearance of the fringe pattern in different sequence of interferograms; this may indicate a step-like relaxing process. The observation challenges linearity of Newton law of cooling since while the system is in cooling down mode and without providing extra energy in the system still higher displacement can be suddenly exhibited, before equilibrium is finally achieved. In general, the tendency to approach equilibrium becomes slower with time. However, there are some puzzling aspects in surface displacement over time. At times the fringe pattern is less dense or may even disappear altogether, only to return later, and suggests that samples are either momentarily stable or that, during dimensional changes, the materials may oscillate about the equilibrium point. However, with the present form of data points (graph in Figure 6), although significant in number and duration of acquisition, the Fourier transform did not reveal any frequency to support an oscillation pattern mechanism. An exponential decay curve may safely represent then this behaviour pattern. The statistical error of ∼1 micron is also high allowing for further future experiments to be drawn for investigation of this discontinuous response pattern.

The way that materials change shape with a change in environment should be studied closely related to ageing. This factor, in combination with repeatable changes, appears to accumulate tension in the structure of the material even when macroscopically it presents little or no dimensional change. The environmental history of the object is related closely to the ultimate safety of changes and should be taken into account.

## 5. Conclusions

The high spatial resolution technique of full-field optical holographic interferometry in a masking double exposure sequence and in a geometrical arrangement with sensitivity to z axis displacement is employed in the laboratory where conditioning control is strict and chosen environmental parameters in a vibration free room can be studied. As an artwork responds to an environmental change to reach equilibrium with the surrounding environment the surface moves in correlation to the surface normal in order to complete the swelling and the shrinking process. By holographically recording the normal to the surface displacement, correlation to the motion of the surface or displacement is achieved. Environmental conditions which may be typically encountered in the exhibition or transport of works of art have been investigated to provoke the dimensional alteration in various materials and a model painting. Data from interferometric images can be acquired from artworks illuminated with pulsed and continuous wave laser over the length of time required to obtain equilibrium. The exposure time is a function of the displacement rate; depending on the magnitude and value of external load the occurred dimensional change exhibits a dynamic variety of responses. Multiple interferograms can be optically acquired with a masking procedure which can be also used as a self indicator of rate of change of the studied material witnessing optimum experimental parameters. Prior to measurement, exposure and time interval between exposures need to be determined for the successful acquisition of interferograms.

The results confirm the importance of assessing the environmental influence directly from the artwork by monitoring distinctively its piece dimensional changes. The strategy foreseen may allow for long term preservation planning according to the safest conditioning dependent on the unique responses of each artwork under concern.

The length of time during which a material responds with a displacement between a pre-set time intervals of exposures can be used to evaluate the effect of changes in environmental conditions on the deterioration or stability of an object. In this way a characteristic reaction in relation to the environmental parameters such as the rate of change in RH can be revealed from the reaction of the materials immediately after the change and during the first hours. By observing this behaviour an immediate profile of the sensitivity is obtained by extrapolation even when the long term distribution of equilibrium moisture content is not achievable. Keeping data of assigned equilibrium time as a response to a constant value of stress taken from experimental data results in a comparative graph describing the material. Thus the graph is an indication of the flexibility of the material and the damage threshold.

The repeatability of holographic interferometry and the rapid acquisition of data associated with the technique, as compared to indirect techniques (for example with strain gauges [7]) are among the main advantages of the technique in the application involved. The application of holographic interferometry can be extended to a variety of artworks that respond dimensionally to environmental variations such as relative humidity (RH) and temperature (T), independently of material composition, surface texture, shape complexity and object dimensions.

Holographic interferometry monitoring represents a non-contacting, non-invasive and non-interventive way to record dimensional change directly from the surface of objects, providing data which indicates the degree of environmental influence. The obtained information is characteristic of the individual material/artwork response, essential for establishing climatic tolerance levels and critical in preventive conservation of individual artworks. Additionally the obtained knowledge is useful to characterise sensitivity levels of individual materials used in artworks enabling correlation from new material to aged masterpieces. Moreover as deformation that might occur in response to small variations in the environment is recorded, each sample's characteristic profile is discovered and can be used to predict long-term patterns of behavior based on short-term non-destructive investigations. Besides, by keeping interferometric fringe data over the years an artifact's fracture or material failure can be foreseen well before its occurrence. Therefore, sensitivity to environmental changes and onset of fatigue or damage can become identifiable and preventable. Additionally the inevitable and undesirable dimensional change which occurs due to induced thermal stress can be used as a dimensional sensor to characterize a baseline value for structural stability under a specific thermal load.

Monitoring of start-up dimensional processes can be used in stimulated samples to extrapolate reaction to specific changes in order to avoid accidental damage since start-up reactions are immediate and intense as the study proves. In this paper a fundamental study on conservation materials dimensional changes was presented. The experiments were performed in laboratory conditions while similar experiments for on-field applications in real environmental open air conditions using portable systems based on holographic digital speckle pattern interferometry are in progress.

## Figures and Tables

**Table 1.** Laser and Environmental parameters.

**Lasers**	**Wavelength**	**Max power output**	**Pulse duration**
Pulsed Ruby	694 nm	10 J	15 ns
Continuous wave Krypton-ion	647 nm	250 mW	20-30 s
Continuous wave Nd:YAG	530 nm	2 W	20-30 s

**Photosensitive recording medium**			
AGFA holotest 8 e 75 hd red-sensitive, bb plates green sensitive, ccd			
**Environmental data recording medium**			
1600 Grant Squirrel meter-logger, type: sq 16–2u / 2l, Range:-10° C– 50°C, 0–95 % RH			

**Environmental control**			
Airtight box: 1 cm thick Perspex in a 30 cm cube equipped with an ultrasonic humidifier, heater, hotair gun and soluble salt solutions			
**Saturated salt solutions**			
Sodium Nitrate (NaNO_3_), 65% RH			
Sodium chloride (NaCl), 75% RH			
Potassium chloride (KCl), 85% RH)			

**Table 2.** Experimental RH loading and unloading sequence scheme.

**Phase**	**Loading/ % RH**	**Unloading/ %RH**	**Rate of Change of RH/%RH.h^-1^**

1	45–65	65–55	3–5
2	55–65	65–45	3–5
3	45–75	75–45	5–10
4	45–85	85–45	10–15
